# NADPH-oxidase 4 gene over-expression in peripheral blood lymphocytes of the schizophrenia patients

**DOI:** 10.1371/journal.pone.0269130

**Published:** 2022-06-13

**Authors:** Elizaveta S. Ershova, Galina V. Shmarina, Andrey V. Martynov, Natalia V. Zakharova, Roman V. Veiko, Pavel E. Umriukhin, George P. Kostyuk, Sergey I. Kutsev, Natalia N. Veiko, Svetlana V. Kostyuk

**Affiliations:** 1 Research Centre for Medical Genetics, Moscow, Russia; 2 N. A. Alexeev Clinical Psychiatric Hospital №1, Moscow Healthcare Department, Moscow, Russia; 3 Normal Physiology Departement, I.M. Sechenov First Moscow State Medical University, Moscow, Russia; School of Pharmacy, Ardabil University of Medical Sciences, ISLAMIC REPUBLIC OF IRAN

## Abstract

**Introduction:**

Increased systemic oxidative stress is common in schizophrenia (SZ) patients. NADPH-oxidase 4 (NOX4) is the cell oxidoreductase, catalyzing the hydrogen peroxide formation. Presumably, NOX4 is the main oxidative stress factor in a number of diseases such as cardiovascular diseases and cancer. We hypothesized that NOX4 may be involved in the oxidative stress development caused by the disease in the schizophrenic patients’ peripheral blood lymphocytes (PBL).

**Materials and methods:**

The SZ group included 100 patients (68 men and 32 women aged 28 ± 11 years). The control group included 60 volunteers (35 men and 25 women aged 25 ± 12 years). Flow cytometry analysis (FCA) was used for DNA damage markers (8-oxodG, ɣH2AX), pro- and antiapoptotic proteins (BAX1 and BCL2) and the master-regulator of anti-oxidant response NRF2 detection in the lymphocytes of the untreated SZ patients (N = 100) and the healthy control (HC, N = 60). FCA and RT-qPCR were used for NOX4 and RNA*NOX4* detection in the lymphocytes. RT-qPCR was used for mtDNA quantitation in peripheral blood mononuclear cells. Cell-free DNA concentration was determined in blood plasma fluorimetrically.

**Results:**

8-oxodG, NOX4, and BCL2 levels in the PBL in the SZ group were higher than those in the HC group (p < 0.001). ɣH2AX protein level was increased in the subgroup with high 8-oxodG (p<0.02) levels and decreased in the subgroup with low 8-oxodG (p <0.0001) levels. A positive correlation was found between 8-oxodG, ɣH2AX and BAX1 levels in the SZ group (p <10^−6^). NOX4 level in lymphocytes did not depend on the DNA damage markers values and BAX1 and BCL2 proteins levels. In 15% of PBL of the HC group a small cellular subfraction was found (5–12% of the total lymphocyte pool) with high DNA damage level and elevated BAX1 protein level. The number of such cells was maximal in PBL samples with low NOX4 protein levels.

**Conclusion:**

Significant *NOX4* gene expression was found a in SZ patients’ lymphocytes, but the corresponding protein is probably not a cause of the DNA damage.

## Introduction

Oxidative stress is an established mediator of aging and age-related disorders including dementia. Elevated local and systemic oxidative stress levels are common features of severe mental illness [1–4]. Under oxidative stress, both nuclear and cell-free DNA undergo oxidative modification. It was shown that schizophrenia is associated with increased systemic nucleic acid damage from oxidation [4]. DNA damage could constitute a molecular link between schizophrenia and accelerated aging [5–12].

8-oxo-2’-deoxyguanosine (8-oxodG), the main product of DNA oxidation, is an important biomarker of in vivo oxidative DNA damage [13, 14]. Post mortem studies have revealed a higher 8-oxodG level in the midbrain, caudate putamen and hippocampus in SZ patients compared to the controls [4, 15]. The main method used for the systemic DNA oxidation level assessement in health and mental illness is the 8-oxodG content analysis in the urine [2, 8, 16] and/or in the blood [16–18]. Our recent studies have shown a significant increase of 8-oxodG content in both peripheral blood lymphocytes (PBL) and circulating cell- free DNA of schizophrenia patients [19, 20].

Some authors suggest the various causes of systemic oxidative stress in the blood and brain cells of SZ patients [15]. We hypothesized that the protein NADPH-oxidase 4 (NOX4) may be involved in maintaining a high ROS level in PBL of the SZ patients. NOX4 is one of the seven members of Nox family (Nox1-5, Duox1 and Duox2). It is a constitutively active enzyme, catalyzing the hydrogen peroxide formation. NOX4 is found primarily on intracellular membranes, on mitochondria, the endoplasmic reticulum or the nucleus. The role of NOX4 is controversial because it may play a damaging or protective role in different diseases [21]. Presumably, NOX4 is the main oxidative stress factor in a number of diseases [22]. Some authors consider NOX4 to be a factor that induces oxidative stress with detrimental consequences for cell function and survival [23, 24]. Accordingly, brain damage is commonly associated with increased NOX4 gene expression and consequent oxidative stress development [25]. Other investigators tend to believe that NOX4 is an essential component of the adaptive response promoting cell survival in an unfavourable environment [26–32].

Alterations in *NOX4* expression in schizophrenia are seemed to be poorly investigated. There is the only mention of *NOX4* RNA levels in the brain cells of deceased schizophrenia patients [33]. The purpose of our study was to obtain the answers to the following questions: (1) Is the level of NOX4 in PBL of the SZ patients changed in comparison with healthy controls? (2) Is there a correlation between NOX4 levels and the markers of DNA damage and apoptosis in SZ PBL? (3) What is the most probable NOX4 protein role in the response of SZ PBL to the endogenous oxidative stress?

In the present study, we analyzed the distribution and the average NOX4 protein levels, DNA damage markers (8-oxodG and γH2AX), as well as BAX1, BCL2 and NRF2 proteins in the PBL of schizophrenia patients and healthy controls. It was found that the SZ PBL contained high NOX4 amounts, but this protein is probably not a cause of DNA damage and cell death.

## Methods

### Subjects

The dataset included 160 individuals (60 HC group, 100 SZ group) inhabiting Moscow and the Moscow region.

#### SZ group

Blood samples were obtained from N.A. Alexeev Clinical Psychiatric Hospital №1 SZ patients. Patients were diagnosed with paranoid SZ (F20.00 or F20.01) according to International Classification of Diseases 10 criteria using structured interviews (Mini-International Neuropsychiatric Interview). Diagnoses were also confirmed pursuant to Diagnostic and Statistical Manual of Mental Disorders, 4th Edition criteria. The SZ group included drug-naïve patients of the first episode (N = 35) and the patients with a chronic form who did not take antipsychotics, at least 2–4 weeks before hospitalization. Psychopathology and functionality of patients were measured according to the Positive and Negative Syndrome Scale (PANSS) [34].

Criteria for not being included in the study were as follows:

1. The presence of concomitant mental disorders, such as dependence on drugs and other psychoactive substances, organic mental disorders of any origin, dementia, and mental retardation;
2. Severe somatic and chronic neurological diseases;
3. Severe acute and chronic somatic diseases preventing the examination, which has caused repeated hospitalizations, loss of work, led to disability, entailed the development of severe complications, such as stroke or heart attack, caused the development of acute or chronic insufficiency of internal organs or body systems, and which may affect the diagnosis, course of mental disorder, as well as the conducted drug therapy.

#### HC group

Peripheral blood samples from the HC-group were collected in the Research Centre for Medical Genetics. The HC-group included volunteers (men and women, Table 1) aged 17 to 51 years. All participants did not have any pronounced hereditary diseases, were mentally healthy, and never sought psychiatric help. This group included employees of the institutions that conducted this study and relatives of patients with non-psychiatric diagnoses observed at the Research Center for Medical Genetics.

**Table 1:** Demographic and clinical measures in the SZ and HC groups.

Index	HC	SZ(all)	SZ(f)*	p**	
**N**	**60**	**100**	**35**		
**Age**	**25±12**	**28.0±11.5**	**24±5**	**>0.1**	***U-test***
**Age of SZ onset**		**19.3±8.5**	**24±5**		
**Age of SZ manifestation**		**22.1±6.1**	**25±7**		
**Gender (men/women)**	**35/25**	**68/32**	**28/7**	**>0.06**	***Fisher’s exact test***
**Never smoked (%)**	**68**	**59**	**54**	**>0.06**	
**More than 20 cigarettes a day (%)**	**12**	**19**	**20**	**>0.15**	
**PANSS (SZ, N = 100)**		**95±32**	**90±21**		
**PANSS SZ(8-oxodG<0.25), N = 64**		**81±25**		**<0.05**	***U-test***
**PANSS SZ(8-oxodG<0.25), N = 36**		**101±33**			

(*) subgroup of primary patients with acute psychosis who were later (after re-hospitalization) diagnosed with schizophrenia.

(**) HC and SZ(all) groups comparison.

The investigation was carried out in accordance with the latest version of the Declaration of Helsinki and was approved by the Independent Interdisciplinary Ethics Committee on Ethical Review for Clinical Studies [Protocol №4 (dated March 15, 2019) for the scientific minimally interventional study «Molecular and neurophysiological markers of endogenous human psychoses»]. All participants signed an informed written consent to participate in the study after the procedures had been completely explained.

### Flow Cytometry Analysis (FCA)

Peripheral blood mononuclear cells (PBMCs) were isolated from 15 mL of blood. PMBCs were separated by centrifuging at 1500 rpm for 30 min using lymphocyte separation media (Histopaque 1077, density e 1.077 g/ml, Sigma). Cells were washed with Phosphate-Buffered Saline (PBS), then centrifuged and resuspended in PBS. To fix the cells, the paraformaldehyde (Sigma) was added at a final concentration of 2% at 37°C for 10 min. Cells were washed three times with 0.5% BSA-PBS and permeabilized with 0.1% Triton X-100 (Sigma) in PBS for 15 min at the room temperature.

Each PMBC sample was analyzed in triplicate. Cells (50x103) were washed three times with 0.5% BSA-PBS and stained with 1 μg/mL FITC—(NOX4, 8-oxodG, ɣH2AX, BAX, NRF2, p53, NF-kB(p65) and NOS2) or PE- BCL2 antibody (Abcam) for 4 h at 4°C in the dark, then again washed thrice with 0.5% Bovine Serum Albumin (BSA) in PBS. To quantify the background fluorescence, we stained a portion of the cells with secondary FITC(PE)-conjugated antibodies. Cells were analyzed at CytoFLEX S (Beckman Coulter). The area of (CD3+) and (CD19+) -cells (lymphocytes) was determined on the SSC-FCS plot. Primary data are presented as median values of the signal. In each experiment, for comparison, we analyzed a control standard sample of lymphocytes from a healthy donor. Relative standard error of the FCA was 4 ± 2%.

### Quantitative analysis of human mitochondrial DNA (mtDNA)

To isolate DNA from PBMCs, we used the standard method described in detail earlier [20]. All reagents for DNA extraction were manufactured by Merck & Co. Inc. Briefly: 5 mL of the solution (2% sodium lauryl sarcosylate, 0.04M EDTA, and 150 μg/mL RNAse A (Sigma, USA)) was added to the fresh PBMCs for 45 min (37°C) and then was treated with proteinase K (200 μg/mL, Promega, USA) for 24 h at 37°C. The lysate samples were extracted with an equal volume of phenol, phenol/chloroform/isoamyl alcohol (25:24:1), and chloroform/isoamyl alcohol (24:1), respectively. DNA was precipitated by adding 1/10 volume of 3M sodium acetate (pH 5.2) and 2.5 volume of ice-cold ethanol. The DNA was collected by centrifugation (10,000 g for 15 min at 4°C), washed with 70% ethanol), and dissolved in water. The final DNA quantification is performed fluorimetrically using the PicoGreen dsDNA reagent by Molecular Probes (Invitrogen, CA, USA). The DNA concentration in the sample is calculated according to a DNA standard curve. We use EnSpire equipment (Finland) at λex = 488nm and λem = 528 nm.

The amount of mtDNA was determined by real time qPCR as described previously [20]. Briefly: serial qPCR assay was established using the StepOnePlus (Applied Biosystems). Each reaction contained 10 μL 2хSYBR Premix Ex Taq (PerfectRealTime^™^, Takara Bio), 2 μL primers (10 μM), and 8 μL of DNA (5 ng/μL) for a final volume of 20 μL. All reactions were performed in duplicates. PCR conditions were 6 min at 95°C initial denaturation, followed by 40 cycles of 30 s of denaturation at 95°C, 15 s of primer annealing at 60°C, and 10 s at 72°C of extension.

The following primers [35] were used (Sintol, Russia):

**F CTTCTGGCCACAGCACTTAAAC**; **R GCTGGTGTTAGGGTTCTTTGTTTT** [Human mitochondrial genome NC_012920 (D-loop) hmito(65)] and **F GCTGGGTAGCTCTAAACAATGTATTCA**;**R CCATGTACTAACAAATGTCTAAAATGG** [Human B2M (accession number M17987) hB2M (95), control].

### Concentration of cfDNA in blood plasma

5 ml of the blood was collected from the peripheral vein with a syringe flushed with heparin (0.1 mL/5 mL) under aseptic conditions. Cells were removed from the blood by centrifugation (460×g). Cellular debris was removed by centrifugation (16,000g, 10 min). DNA was isolated from plasma by the standard method [36]. Briefly, 3 mL of plasma were mixed with 0.3 mL of the solution containing 1% sodium lauryl sarcosylate, 0.02 M EDTA, and 75 μg/mL RNAse A (Sigma, USA), incubated for 45 min, then were treated with proteinase K (200 μg/mL, Promega, USA) for 24 h at 37°C. After two cycles of the purification with saturated phenolic solution, DNA fragments were precipitated by adding two volumes of ethanol in the presence of 2 M ammonium acetate. The precipitate was then washed with 75% ethanol twice and dissolved in water. The DNA quantification is performed fluorimetrically using the PicoGreen dsDNA quantification reagent by Molecular Probes (Invitrogen, CA, USA). We use EnSpire equipment (Finland) at excitation and emission wavelengths of 488 and 528 nm, respectively. Relative standard error of the index cfDNA was 10 ± 4%.

### Statistical analysis

All results reported here were reproduced at least two times as independent biological replicates. The average marker level in the cell population was estimated by the FL-signal median value. In the graphs, each point is the mean for three FL-signal median values (three parallel measurements for the same PBMC sample). The significance of the observed differences was analyzed using the non-parametric Mann–Whitney test (p) and Kolmogorov–Smirnov test (D and α). Spearman analysis and linear regression analysis were used to analyze correlations between two parameters. Data were analyzed with StatPlus2007 professional software (http://www.analystsoft.com/). All p-values were two-sided and considered statistically significant at p < 0.05.

MedCalc (https://www.medcalc.org/manual/roc-curves.php) creates a complete sensitivity/specificity report [Receiver Operating Characteristic (ROC) curve analysis]. Each point on the ROC curve represents a sensitivity/specificity pair corresponding to a particular decision threshold. The Area Under the ROC curve (AUC) reflects the parameter difference between two groups (disease/health).

## Results

**Fig 1:**
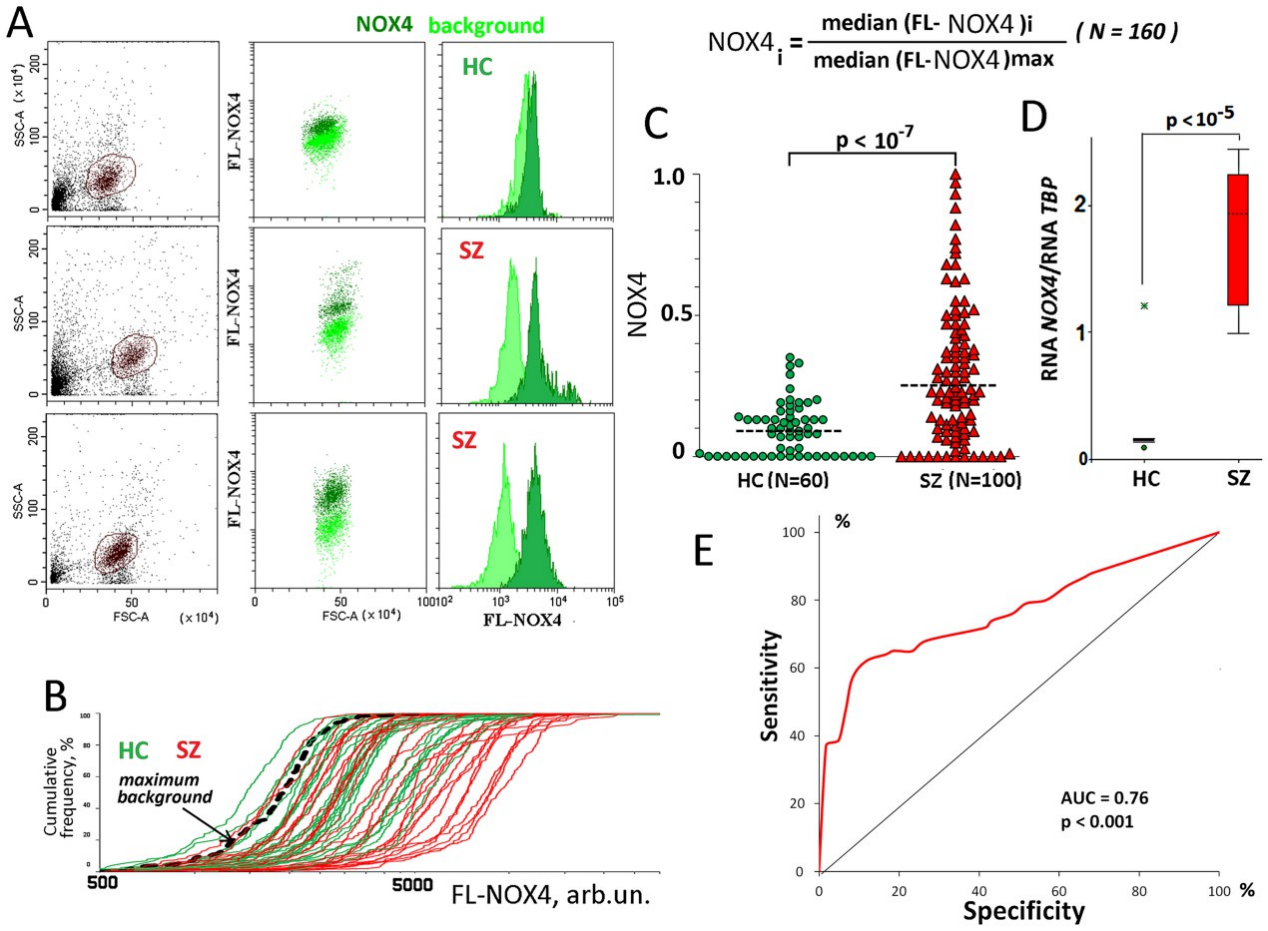
NOX4 levels in the PBL of the SZ and HC groups. A. The most typical examples of the NOX4 analysis in lymphocytes. B. Cumulative distribution of FL1- NOX4 for 63 randomly selected HC (green) and SZ (red) samples in a one experiment. The dashed curve is the background curve for the sample with the maximum background level. C. NOX4 levels in the lymphocyte of the SZ and HC groups. The average level of marker in the cell population was estimated by the FL-signal median value. In the graphs, each point is the mean for three FL-signal median values (three parallel measurements for the same PBMC sample). The median values were normalized to the maximum signal medians values in the analyzed total sample (N = 160). The relative standard error was 7 ± 3%. The significance of the observed differences was analyzed using non-parametric Mann–Whitney (p) test. D. RNA*NOX4* levels determined in lymphocytes of 30 SZ patients and 30 HC. E. ROC curve for the SZ/HC groups. Each point on the ROC curve represents a sensitivity/specificity pair. The area under the ROC curve (AUC) is a measure of how well a parameter can distinguish between two groups (SZ/HC).

We analyzed the isolated PMBCs and blood plasma of the healthy control (HC group, N = 60) and untreated SZ patients (SZ group, N = 100). Clinical and demographic characteristics of the HC group and SZ group are shown in the Table 1. The population of PMBCs isolated from the blood contains several subpopulations of cells (Fig 1A, plots FCS—SSC). PBMCs include lymphocytes, monocytes, and dendritic cells. The cells vary significantly in size and number, which complicates the analysis of the entire PBMCs population by flow cytometry. Therefore, we analyzed only one subpopulation of cells containing lymphocytes (PBL). In humans, the frequencies of these populations vary across individuals, but typically, lymphocytes are in the range of 70–90% [37].

Firstly, we determined the protein NOX4 content in PBL of the HC group and SZ group. Secondly, we compared NOX4 levels with the markers levels reflecting DNA damage and cell death.

### PBL of the untreated SZ patients express increased amounts of NOX4

Fig 1A shows three most typical examples of the cell distribution in PBL populations according the NOX4 level. Fig 1B illustrates the cumulative distributions of FL-NOX4 signals for SZ and HC lymphocytes analyzed in one experiment.

The average NOX4 level in PBL population was estimated by the FL-NOX4 median value. For convenience, the median values were normalized to the maximum median value in the analyzed total (N = 160) sample (**NOX4** index). The data for the two groups are shown in Fig 1C. NOX4 level in the SZ group significantly exceeds the NOX4 level in the HC group (p<10^−7^, U-test). ROC analysis data confirm significant differences between the two groups (Fig 1E).

RNA *NOX4* levels in 30 randomly selected PBL samples of patients and healthy controls were determined (Fig 1D). The level of RNA *NOX4* in the cells of SZ patients significantly exceeded the level of RNA in the cells of the control group.

Combined analysis of RNA and protein levels suggested significant increase of *NOX4* gene expression in lymphocytes of the SZ patients. To characterize a hypothetical correlation between high ROS-producing protein NOX4 levels and the cellular DNA oxidation, we determined 8-oxodG marker levels in PBL of the HC and SZ groups.

### Increased DNA oxidation level in lymphocytes of the untreated SZ patients

**Fig 2:**
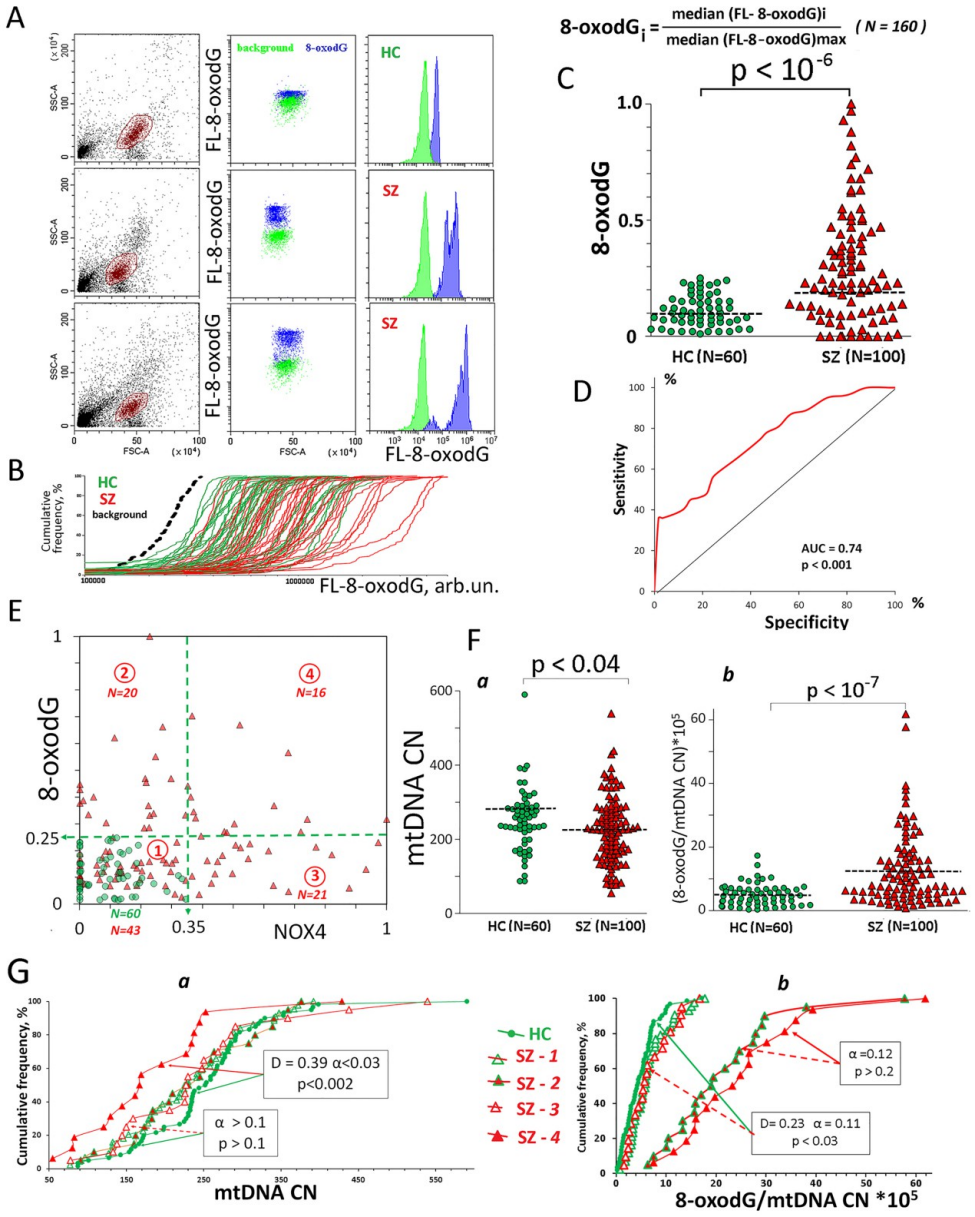
8-oxodG levels in the PBL of the SZ group and HC group. A. The most typical examples of the 8-oxodG analysis in lymphocytes. B. Cumulative distribution of FL1-8-oxodG for 103 randomly selected HC (green) and SZ (red) samples in a single experiment. The dashed curve is the background curve for the sample with the maximum background level. C. **8-oxodG** levels in the lymphocyte of the SZ- and HC groups. The average level of marker in the cell population was estimated by the FL-signal median value. In the graphs, each point is the mean for three FL-signal median values (three parallel measurements for the same PBMC sample). The median values were normalized to the maximum signal medians values in the analyzed total sample (N = 160). The relative standard error was 7 ±2%. The significance of the observed differences was analyzed using non-parametric Mann–Whitney (p) test. D. ROC curve. Each point on the ROC curve represents a sensitivity/specificity pair. The area under the ROC curve (AUC) is a measure of how well a parameter can distinguish between two groups (SZ/HC). E. The dependence of **8-oxodG** on **NOX4**. Dotted lines indicate areas of points included in the SZ subgroups. F. (a) MtDNA CN in the HC and SZ groups. (b) Ratio **8-oxodG/mtDNA CN** for the HC and SZ groups. G. (a) Cumulative distribution of mtDNA CN for HC and SZ subgroups. (b) Cumulative distribution of ratio **8-oxodG/mtDNA CN** for HC and SZ subgroups. Arrows indicate compared subgroups. The significance of the observed differences was analyzed using the non-parametric Mann–Whitney test (p) and Kolmogorov–Smirnov test (D and α).

Fig 2A shows three most typical examples of the cells distribution in PBL populations according the 8-oxodG level. Fig 2B illustrates the cumulative distributions of FL-8-oxodG signals for SZ and HC lymphocytes analyzed in a one experiment.

Relatively low 8-oxodG levels were found in all HC lymphocytes of the population. 8-oxodG content heterogeneity was observed only in 9 (15%) of the HC group samples. A small fraction of cells (5–12% of the total population) with a very high 8-oxodG level compared to the rest of the population was distinguished in these PBL. Single cells with very high 8-oxodG level were also found in the remaining HC samples, but their total content did not exceed 5%.

In SZ-lymphocytes samples, we observed a different pattern of cell distribution according to the oxidation marker content (Fig 2B). 8-oxodG level range significantly increased in some samples of the SZ group. Along with the total marker level increase, two cell subfractions with different 8-oxodG levels were observed. For 20% of the samples, we detected a significant marker content disproportion in the cells. In these samples, 10–20% of the cells contained low marker amounts, and the remaining cells contained large 8-oxodG amounts.

The average level of 8-oxodG in the lymphocyte population was estimated by the FL-8-oxodG median value. For convenience, the median values were normalized to the maximum median value in the analyzed total (N = 160) sample (**8-oxodG** index). **8-oxodG** values for 160 lymphocytes samples are shown in Fig 2C. HC and SZ groups significantly differ by the distribution of the **8-oxodG** (α<10^−4^) and by the **8-oxodG** value (p<10^−6^). Fig 2D provides the ROC analysis data, which confirm significant **8-oxodG** index differences between the two groups.

Fig 2E shows the dependence of the **8-oxodG** index on the **NOX4** index for the 160 PBL. We found no significant correlation between the two parameters in the HC and SZ groups (Table 2 and Fig 2E).

**Table 2:** Multiple-variable analysis (R and p) for SZ and HC groups.

SZ (n = 100)	8-oxoDG	yH2AX	yH2AX(R)	BAX	BCL2	BAX/BCL2	NRF2	mtDNA	CfDNA
NOX4	0.05	0.17	-0.10	0.09	0.22	0.13	0.27	0.03	0.21
	0.6	0.1	0.3	0.4	0.03	0.2	0.01	0.8	0.03
8-oxoDG		0.56	0.57	0.50	-0.09	0.36	0.29	-0.06	0.18
		0.0000	0.0000	0.0000	0.4	0.0000	0.003	0.5	0.1
yH2AX			0.34	0.41	0.01	0.42	0.28	0.02	0.12
			0.001	0.0000	1.0	0.0000	0.01	0.8	0.2
yH2AX(R)				0.24	-0.05	0.19	0.06	0.00	0.06
				0.02	0.6	0.1	0.6	1.0	0.6
BAX					-0.08	0.60	0.33	-0.11	0.21
					0.4	0.0000	0.001	0.3	0.04
BCL2						-0.05	0.12	0.11	0.05
						0.0000	0.2	0.3	0.6
BAX/BCL2							0.10	-0.12	0.11
							0.3	0.2	0.3
NRF2								-0.11	0.17
								0.3	0.1
mtDNA									-0.22
									0.03
HC	8-oxoDG	yH2AX	yH2AX(R)	BAX	BCL2	BAX/BCL2	NRF2	mtDNA	CfDNA
NOX4	-0.14	0.04	0.01	-0.28	-0.02	-0.27	0.04	-0.15	-0.20
	0.3	0.8	0.9	0.0	0.9	0.0	0.8	0.3	0.1
8-oxoDG		0.19	0.23	0.34	0.03	0.153	0.16	0.01	0.03
		0.2	0.1	0.01	0.9	0.244	0.2	0.9	0.8
yH2AX			0.04	0.16	-0.15	0.144	-0.027	0.30	0.08
			0.8	0.2	0.2	0.274	0.84	0.02	0.6
yH2AX(R)				0.15	0.09	-0.02	-0.05	0.05	0.12
				0.2	0.5	0.9	0.7	0.7	0.4
BAX					-0.02	0.72	-0.30	0.17	0.09
					0.9	0.0000	0.02	0.2	0.5
BCL2						-0.52	0.02	-0.18	0.01
						0.0000	0.9	0.2	0.9
BAX/BCL2							-0.28	0.37	0.06
							0.03	0.004	0.6
NRF2								0.04	-0.10
								0.8	0.5
mtDNA									0.04
									0.7

Yellow and blue color: p<0.05 и p<0.005

Brown and dark blue: p<0.0001

In order to understand the possible role of NOX4 in the DNA oxidation in PBL, we divided the SZ group into four subgroups (Fig 1E):

SZ-1 (NOX4 < 0.35; 8-oxodG< 0.25; N = 43)—the NOX4 and 8-oxodG ranges coincide with the range in the HC group;
SZ-2 (NOX4 <0.35; 8-oxodG> 0.25; N = 20)–the 8-oxodG range is higher than in the HC group;
SZ-3 (NOX4 >0.35; 8-oxodG<0.25; N = 21)—the NOX4 range is higher than in the HC group;
SZ-4 (NOX4 >0.35; 8-oxodG>0.25; N = 16)—the NOX4 and 8-oxodG ranges are higher than in the HC group;

### HC (8-oxodG< 0.25; NOX4 <0.35; N = 60)

43% of the SZ patients contained in PBL the amounts of NOX4 and 8-oxodG comparable to the amounts of these markers in the control group. 16% of the PBL in the SZ group contained large amounts of both markers. We found no correlation between the **NOX4** and **8-oxodG** in any of the subgroups (p>0.1).

### Oxidative modification of mitochondrial and nuclear DNA

8-oxodG marker reflects the total oxidized dG nucleoside level in the cellular DNA. Mitochondrial DNA (mtDNA) is highly susceptible to oxidative damage due to lack of protective histones and limited DNA repair capacity [38]. It can be assumed, that the mtDNA, which is more oxidized than the nuclear DNA, produces the main contribution to the FL- signal value.

We compared the levels of **8-oxodG** and mtDNA copy number (mtDNA CN) in the PBL (Fig 2F*a* and 2F*b*). The cells of the SZ patients contained lower mtDNA CN than the cells of the HC group (Fig 2F*a*). However, a more detailed analysis showed that the decrease in the mtDNA CN occurs only in the SZ-4 subgroup (Fig 2G*a*).

The ratio reflecting the amount of 8-oxodG per one copy of mtDNA for the SZ group was significantly higher than for the HC group (Fig 2F*b*). The PBL in the SZ-2 and SZ-4 groups expressed higher ratio r = **8-oxodG**/mtDNA CN than the PBL in the HC group (Fig 2G*b*).

For the control group, we found a strong positive correlation between the ratio **r** and **8-oxodG** (Rs = 0.84, p = 0). In the SZ- (8-oxodG<0.25) subgroups (SZ-1 and SZ-3), the positive correlation between the ratio **r** and **8-oxodG** was weaker (Rs = 0.71, p≤0.0001). In the SZ(8-oxodG>0.25) subgroups (SZ-2 and SZ-4), there was no positive correlation between **r** and **8-oxodG** (p>0.1).

These data may indirectly indicate that not only mtDNA is oxidized in the SZ-2 and SZ-4 subgroups, but also nuclear DNA.

To confirm the high DNA damage level in the cell nuclei of some SZ patients, we used the DNA double-strand break analysis using antibodies to Phosphorylated on serine 139 histone H2AX (ɣ-H2AX).

### Different DNA damage levels in cell nuclei of the SZ subgroups

**Fig 3:**
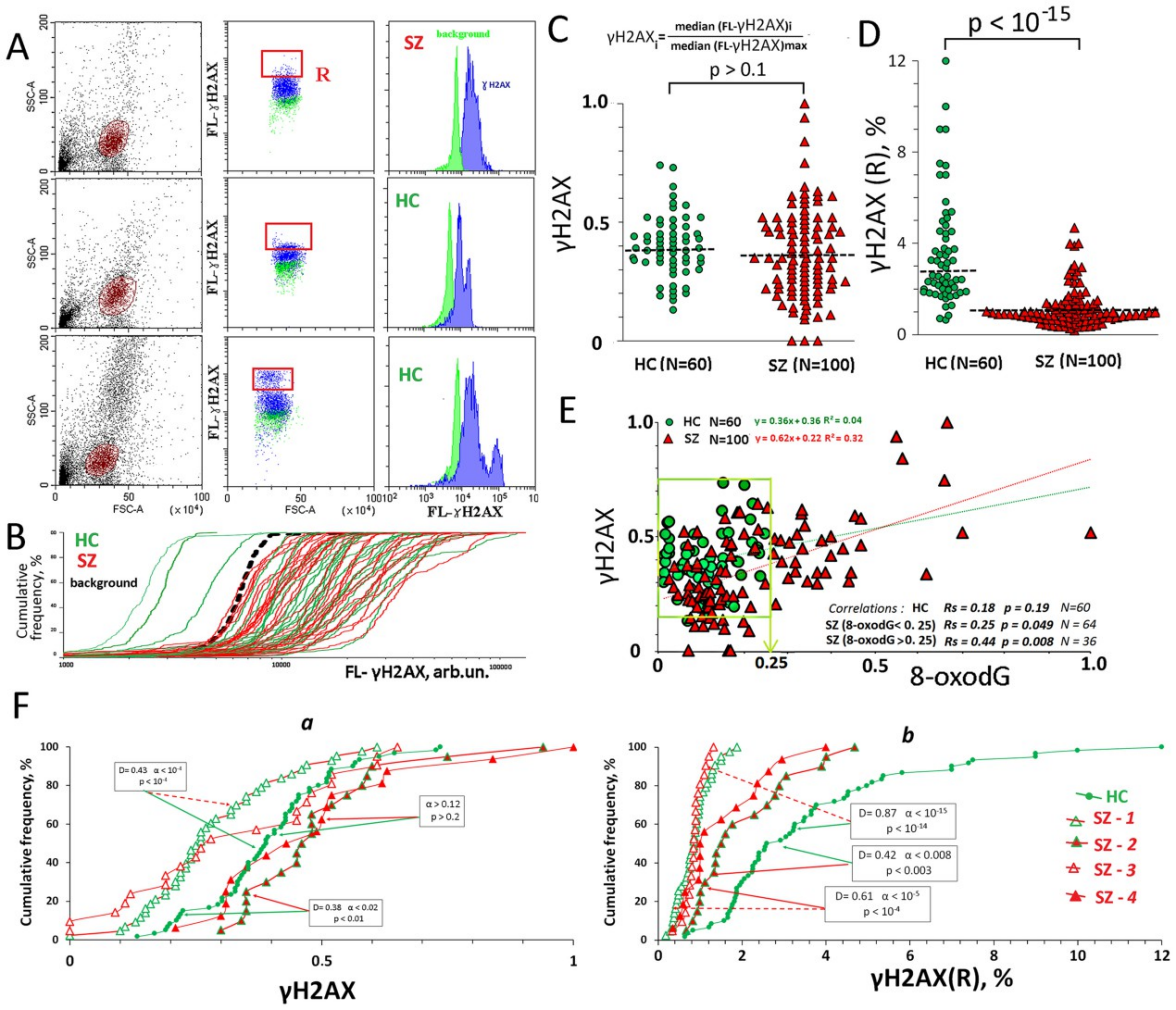
ɣH2AX levels in the lymphocyte of the SZ group and HC group. A. The most typical examples of the ɣH2AX analysis in lymphocytes. B. Cumulative distribution of FL1- ɣH2AX for 60 randomly selected HC (green) and SZ (red) samples in a one experiment. The dashed curve is the background curve for the sample with the maximum background level. C. ɣH2AX levels in the lymphocyte of the SZ- and HC groups. The average level of marker in the cell population was estimated by the FL-signal median value. In the graphs, each point is the mean for three FL-signal median values (three parallel measurements for the same PBMC sample). The median values were normalized to the maximum signal medians values in the analyzed total sample (N = 160). The relative standard error was 5 ± 3%. The significance of the observed differences was analyzed using non-parametric Mann–Whitney (p) test. D. **ɣH2AX(R)** levels in the lymphocyte of the SZ- and HC groups. The significance of the observed differences was analyzed using non-parametric Mann–Whitney (p) test. E. The dependence of **ɣH2AX** on **8-oxodG**. Linear regression data is shown at the top left of the graph. At the bottom right of the graph, italics show the correlation analysis data for the SZ subgroups and HC group. F. (a) Cumulative distribution of **ɣH2AX** for HC and SZ subgroups. (b) Cumulative distribution of **ɣH2AX(R)** for HC and SZ subgroups. Arrows indicate compared subgroups. The significance of the observed differences was analyzed using the non-parametric Mann–Whitney test (p) and Kolmogorov–Smirnov test (D and α).

Fig 3A(1) shows the typical examples of the γH2AX analysis by the FCA. Fig 3B illustrates the cumulative signal distributions for PBL that were analyzed in a one experiment. Both groups included PBL with low and high γH2AX protein content. During the analysis we isolated a γH2AX(R) fraction for each PBL sample, which differs significantly from the rest of the cellular population by the marker level (indicated with red color in Fig 3A). 9 (15%) of the HC samples contained from 5 to 12% of cells with abnormally high γH2AX levels compared to the rest of the cells in this population. PBL of the HC group contained significantly higher γH2AX (R) cells than PBL of the SZ subgroups (Fig 3D and 3F*b*).

The average γH2AX level in the cell population was characterized by the normalized median of the signal values (**γH2AX** index). The data for the two groups are shown in Fig 3C. The distributions and the levels of the **γH2AX** values in the HC and SZ groups did not differ (α > 0.01; p> 0.1). However, for the SZ subgroups that differ by the **8-oxodG** we found significant differences (Fig 3F). SZ(8-oxodG<0.25) samples (SZ-1 and SZ-3 subgroups) contained significantly lower γH2AX amounts than the SZ(8-oxodG>0.25) samples (SZ-2 and SZ-4) (p < 10^−5^) and HC samples (p < 10^−3^). The γH2AX level in the SZ(8-oxodG>0.25) subgroups was higher than in the control group (p< 0.02).

For the SZ group (N = 100), there was a positive correlation (Rs = 0.56, p = 10^−8^) between the **8-oxodG** and **γH2AX**, which reflect the DNA damage level [Table 2 and Fig 3E]. In the SZ(8-oxodG>0.25) subgroups, the positive correlation was much stronger (p = 0.008) than in the SZ(8-oxodG<0.25) subgroups (p = 0.049) and HC group (p>0.1). A positive correlation between the **8-oxodG** and **γH2AX** in the subgroups SZ(8-oxodG>0.25) confirms the oxidation of nuclear DNA in the PBL of this SZ subgroup.

High DNA damage level in the cells might stimulate cell apoptosis. To test this assumption, we analyzed pro-apoptotic protein BCL2 Associated X (BAX) and anti- apoptotic protein B-cell lymphoma 2 (BCL2) level in lymphocytes.

### Increased level of the apoptosis markers in the SZ(8-oxodG>0.25) subgroups

BAX functions as an apoptotic activator. The protein has been shown to be involved in p53-mediated apoptosis [39]. Protein BCL2 regulates cell death inhibiting apoptosis [40].

#### BAX

**Fig 4:**
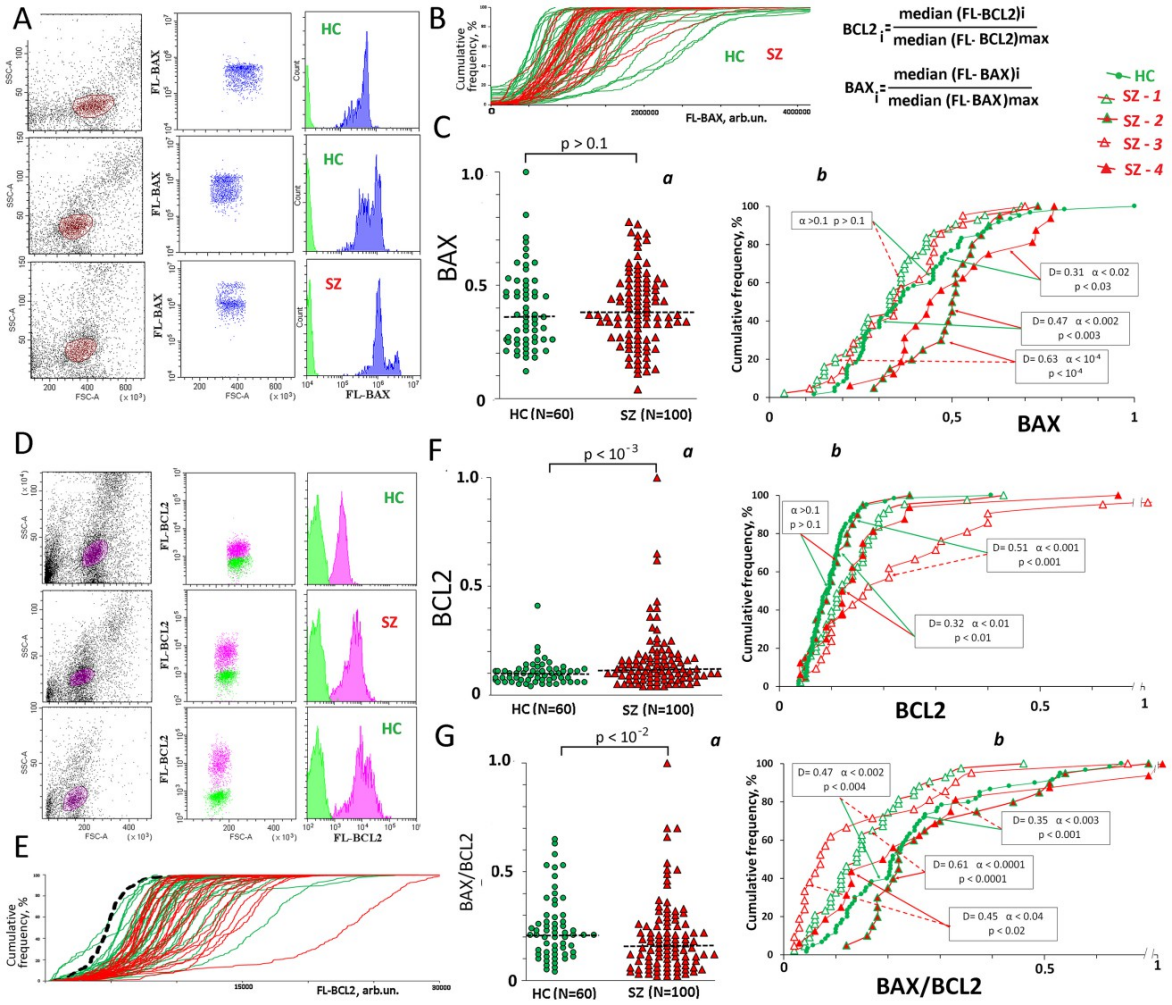
BAX and BCL2 levels in the lymphocyte of the SZ and HC groups. A. The most typical examples of the BAX analysis in lymphocytes. B. Cumulative distribution of FL1- BAX for 80 randomly selected HC (green) and SZ (red) samples in a one experiment. C. BAX levels in the lymphocyte of the SZ and HC groups. (a) The average level of marker in the cell population was estimated by the FL-signal median value. In the graphs, each point is the mean for three FL-signal median values (three parallel measurements for the same PBMC sample). The median values were normalized to the maximum signal medians values in the analyzed total sample (N = 160). The relative standard error was 8 ± 1%. The significance of the observed differences was analyzed using non-parametric Mann–Whitney (p) test. (b) Cumulative distribution of **BAX** for HC and SZ subgroups. D. The most typical examples of the BCL2 analysis in lymphocytes. E. Cumulative distribution of FL1- BAX for 60 randomly selected HC (green) and SZ (red) samples in a one experiment. The dashed curve is the background curve for the sample with the maximum background level. F. (a) BCL2 levels in the lymphocyte of the SZ and HC groups. The average level of BCL2 in the cell population was estimated by the FL-signal median value. In the graphs, each point is the mean for three FL-signal median values (three parallel measurements for the same PBMC sample). The relative standard error was 4 ± 2%. (b) Cumulative distribution of **BCL2** for HC and SZ subgroups. G. (a) Ratio BAX/BCL2 for the SZ and HC groups. (b) Cumulative distribution of **BAX/BCL2** for HC and SZ subgroups. Arrows indicate compared subgroups. The significance of the observed differences was analyzed using the non-parametric Mann–Whitney test (p) and Kolmogorov–Smirnov test (D and α).

Fig 4A shows the typical examples of the BAX levels analysis. Fig 4B shows the cumulative signal distributions for lymphocyte samples that were analyzed in a one experiment. As a rule, two subpopulations of cells are distinguished in the lymphocytes of both groups—with high and low BAX protein levels. Fig 4C*a* shows the normalized medians of the signal values (**BAX** index) for the SZ and HC groups. **BAX** values and distributions for HC- and SZ groups do not differ significantly. For SZ group, we found a positive correlation between the DNA damage markers (8-oxodG and γH2AX) and **BAX** (Table 2).

The Fig 4C*b* shows a comparison of protein BAX levels in SZ subgroups, which differ by the level of NOX4 and DNA oxidation. The BAX level in the subgroups SZ(8-oxodG>0.25) was higher than the BAX level in the control group (p< 0.004) and in the SZ(8-oxodG<0.25) subgroups (p < 10^−4^).

#### BCL2

In contrast to the BAX, BCL2 protein distribution in the cells was broader, and the cells division into the fractions was observed only for single samples (Fig 4D and 4E). Fig 4F*a* shows the normalized signal values medians distributions (**BCL2** index) for the SZ and HC groups. BCL2 protein content in the lymphocytes of patients was significantly higher than in the healthy people lymphocytes. We found no significant correlation between DNA damage and BCL2 levels (Table 2).

The Fig 4F*b* shows a comparison of protein BCL2 levels in SZ subgroups, which differ by the DNA oxidation and NOX4 protein levels. The BCL2 level in the subgroup SZ-3 (8-oxodG<0.25; NOX4 >0.35) was higher than the BCL2 level in the control group (p< 0.001) and other SZ-subgroups.

Fig 4G*a* shows BAX/BCL2 ratio, allowing to estimate the number of cells in a population that are potentially able induce apoptosis [41]. In the HC group, BAX/BCL2 ratio was higher than in the SZ group (p<0.01). In the HC group and in the SZ(8-oxodG>0.25) subgroups, BAX/BCL2 ratio was higher than in the SZ(8-oxodG<0.25) subgroups (p< 0.0001, Fig 4G*b*).

#### Сell-free DNA concentration

With regard to cell apoptosis in schizophrenia, conflicting data are reported in the literature. Some authors found an increase in the apoptosis, the others demonstrated impaired cell apoptosis in the schizophrenia patients [19, 42–45]. The lower BAX/BCL2 ratio values in the SZ(8-oxodG<0.25) subgroups (Fig 4G*b*) indicate possibly lower apoptosis level in the PBL. However, the result of lymphocytes apoptosis level determination depends on the apoptotic cells elimination rate from the population, especially if apoptosis is analyzed not in the whole blood, but in isolated cells. Increased damaged cells clearance from the population may lead to an underestimation of the apoptotic and necrotic cells number in the sample [46]. The overall level of cell death in patients with schizophrenia can be estimated by the blood plasma cfDNA concentration analysis [47].

**Fig 5:**
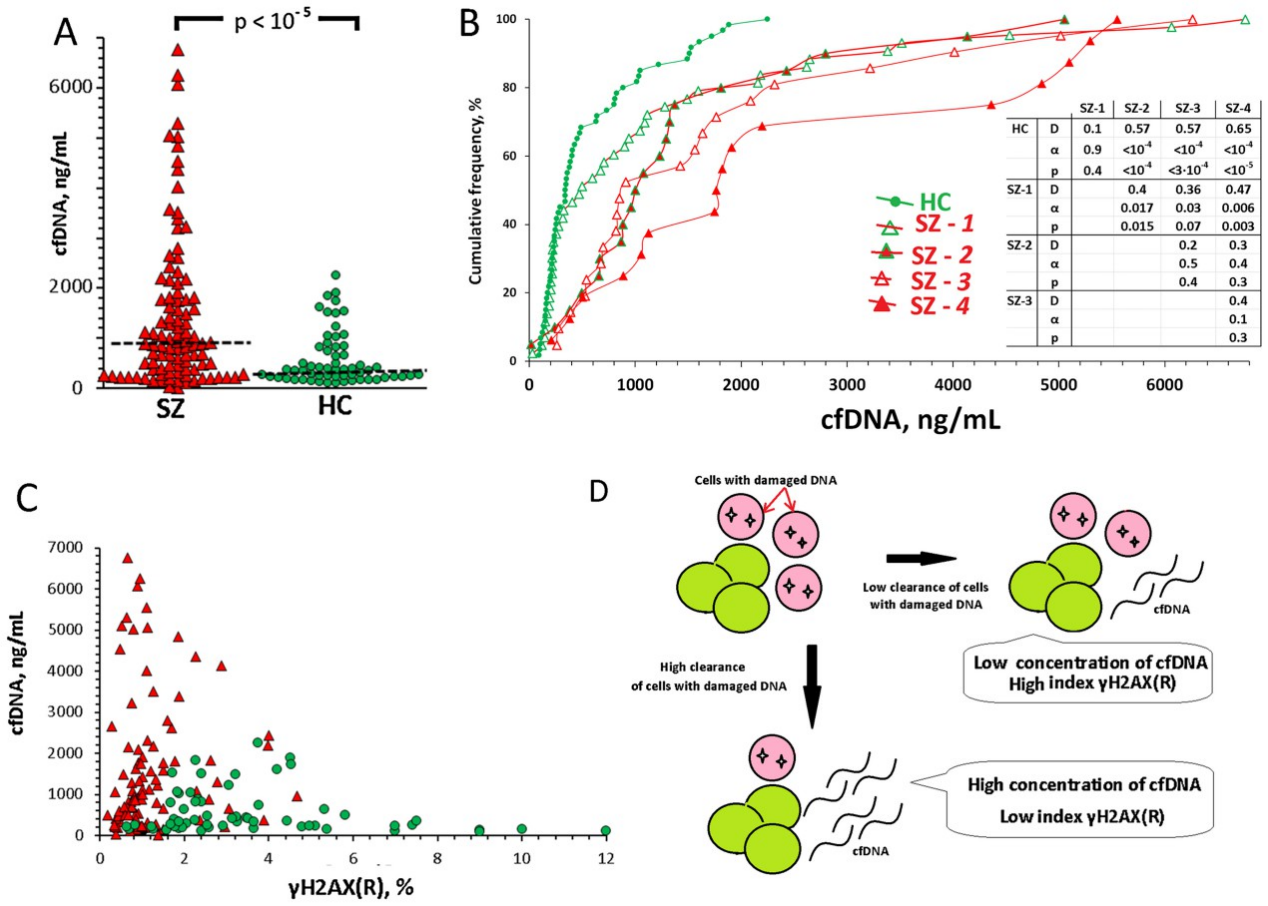
Concentration of the circulating cell-free DNA (cfDNA) in blood plasma. A. Concentration of the cfDNA in SZ- and HC groups. B. Cumulative distribution cfDNA for HC and SZ subgroups. The significance of the observed differences was analyzed using the non-parametric Mann–Whitney test (p) and Kolmogorov–Smirnov test (D and α). C. Dependence of cfDNA concentration in blood plasma on the size of lymphocyte fraction γH2AX (R) with damaged DNA. D. Scheme illustrating the change in the ratio of the size of the cell fraction γH2AX (R) and the concentration of cfDNA.

Data on the cfDNA concentration in HC and SZ groups are given in Fig 5A. In the blood plasma of patients, cfDNA concentrations (15–6700 ng/mL, median 926 ng/mL) are significantly higher (p<0.00001) than in the blood plasma of HC-group (93–2200 ng/mL, median 346 ng/mL), which coincides with the data of other authors [Jiang et al., 2018]. This fact reflects a higher cell death level in the schizophrenia patients. The cfDNA level in the subgroups SZ(NOX4>0.35) was higher (p<0.04) than the cfDNA level in the SZ(NOX4<0.35) subgroups (Fig 5B). Comparison of the subgroups SZ-1 and SZ-2 or SZ-3 and SZ-4 showed that higher cfDNA concentrations are associated with higher levels of 8-oxodG in isolated lymphocytes. The maximum cfDNA concentrations were observed in the plasma of patients in the subgroup SZ-4 (8-oxodG>0.25; NOX4 >0.35).

#### Nuclear factor erythroid 2-related factor 2 (NRF2)

It is known that NOX4 contributes to antioxidant response through regulation of *NFE2L2* gene expression and the activity of Nuclear factor erythroid 2-related factor 2 (NRF2) in the cells exposed to endogenous and exogenous damaging factors [48].

**Fig 6:**
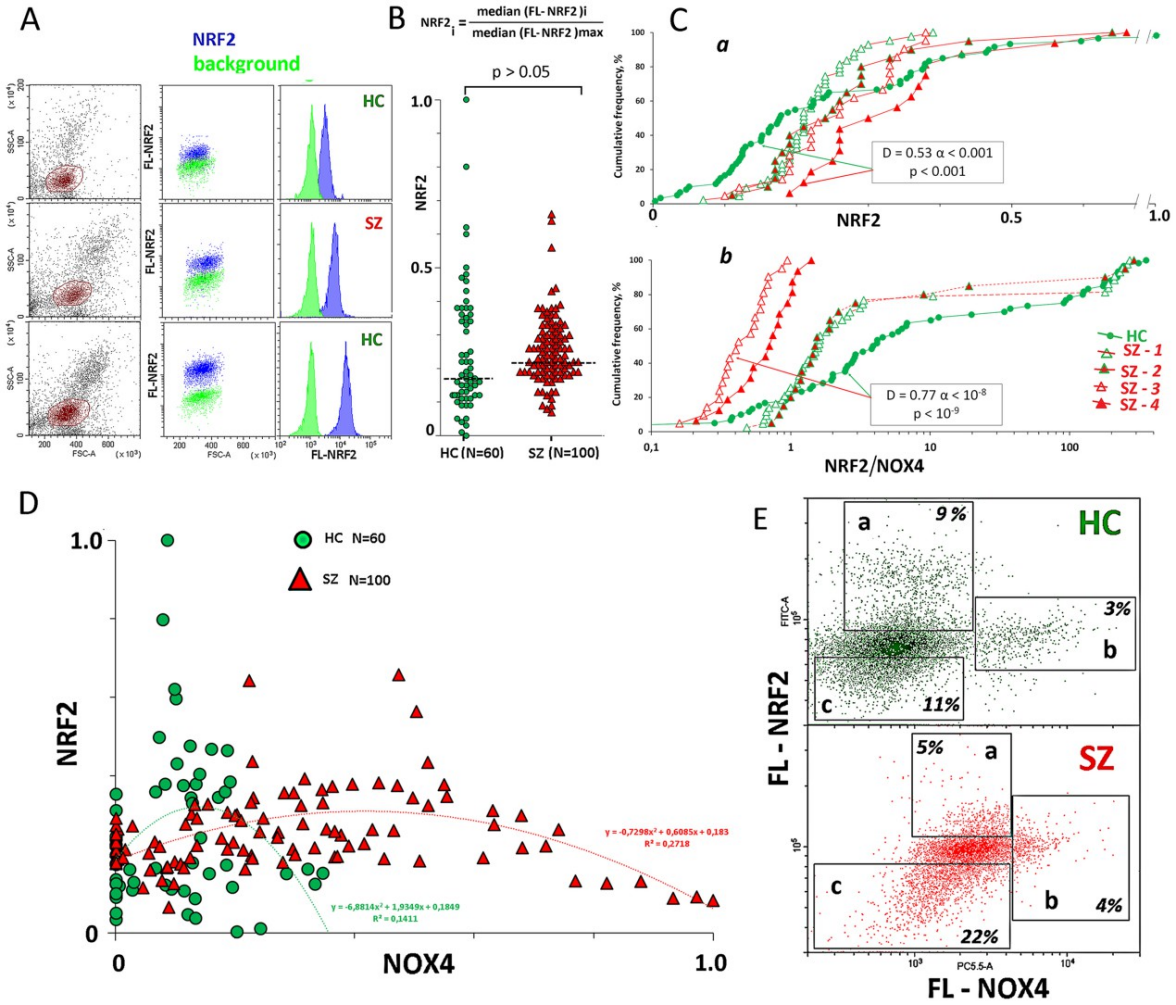
NRF2 levels in the lymphocyte of the SZ and HC groups. A. The most typical examples of the NRF2 analysis in lymphocytes. B. NRF2 levels in the lymphocyte of the SZ and HC groups. The average level of marker in the cell population was estimated by the FL-signal median value. In the graphs, each point is the mean for three FL-signal median values (three parallel measurements for the same PBMC sample). The median values were normalized to the maximum signal medians values in the analyzed total sample (N = 160). The relative standard error was 5 ± 2%. The significance of the observed differences was analyzed using non-parametric Mann–Whitney (p) test. C. (a) Cumulative distribution of **NRF2** for HC and SZ subgroups. (b) Cumulative distribution of ratio **NRF2/NOX4** for HC and SZ subgroups. D. The dependence of **NRF2** on **NOX4** for the SZ and HC groups. E. Lymphocyte [HC (green) and SZ (red)] staining with two types of antibodies with different labels: NRF2(FITC) and NOX4(PC5.5).

Fig 6A shows the typical examples of NRF2 levels analysis by the FCA. The total NRF2 level was characterized by the normalized median of the signal values (**NRF2** index). The HC- and SZ groups did not differ by the NRF2 protein levels (Fig 6B). However, SZ-4 subgroup (8-oxodG>0.25; NOX4 >0.35) significantly differed from all other SZ subgroups and HC group by high NRF2 content (Fig 6C*a*).

The dependence of **NRF2** on **NOX4** was analyzed (Fig 6D). For both groups, the maximum **NRF2** values were observed at average **NOX4** values. The levels of NOX4 and NRF2 proteins in double staining PBL samples of patients (n = 10) and healthy controls (n = 10) were assesed. The most typical examples are shown in Fig 6E. We observed the similar patterns both in the patients and control groups: the cells with exremely high NRF2 protein levels [fraction (a)] had average NOX4 protein levels; the cells with extremely high NOX4 protein levels [fraction (b] contained decreased amounts of NRF2. In addition, each PBL sample included cell fraction with abnormally low levels of NRF2 (fraction (c)). Thus, the level of the NRF2 protein was nonlinearly dependent on the NOX4 protein level.

In the SZ(NOX4>0.35) subgroups with high NOX4 level, the ratio NRF2/NOX4 is significantly lower than in the HC and SZ(NOX4<0.35) subgroups, despite the increased NRF2 level in the SZ-4 subgroup (Fig 6C*b*).

#### γH2AX(R) subpopulation analysis

We found it unusual to find in some HC-lymphocytes populations γH2AX(R) fraction with abnormally high **γH2AX, BAX** and **BAX/BCL2** indices against the background of low average markers values. At the same time, the SZ-lymphocyte populations contained fewer cells of the γH2AX(R) fraction against the background of much higher average **8-oxodG** and **NOX4** values. To understand the reasons for such dissonance we additionally analyzed the γH2AX(R) subpopulation of cells in 10 samples of the HC group with high γH2AX(R) fraction (5–12%) and in 10 HC group cells samples with low γH2AX(R) fraction (less than 3%).

**Fig 7:**
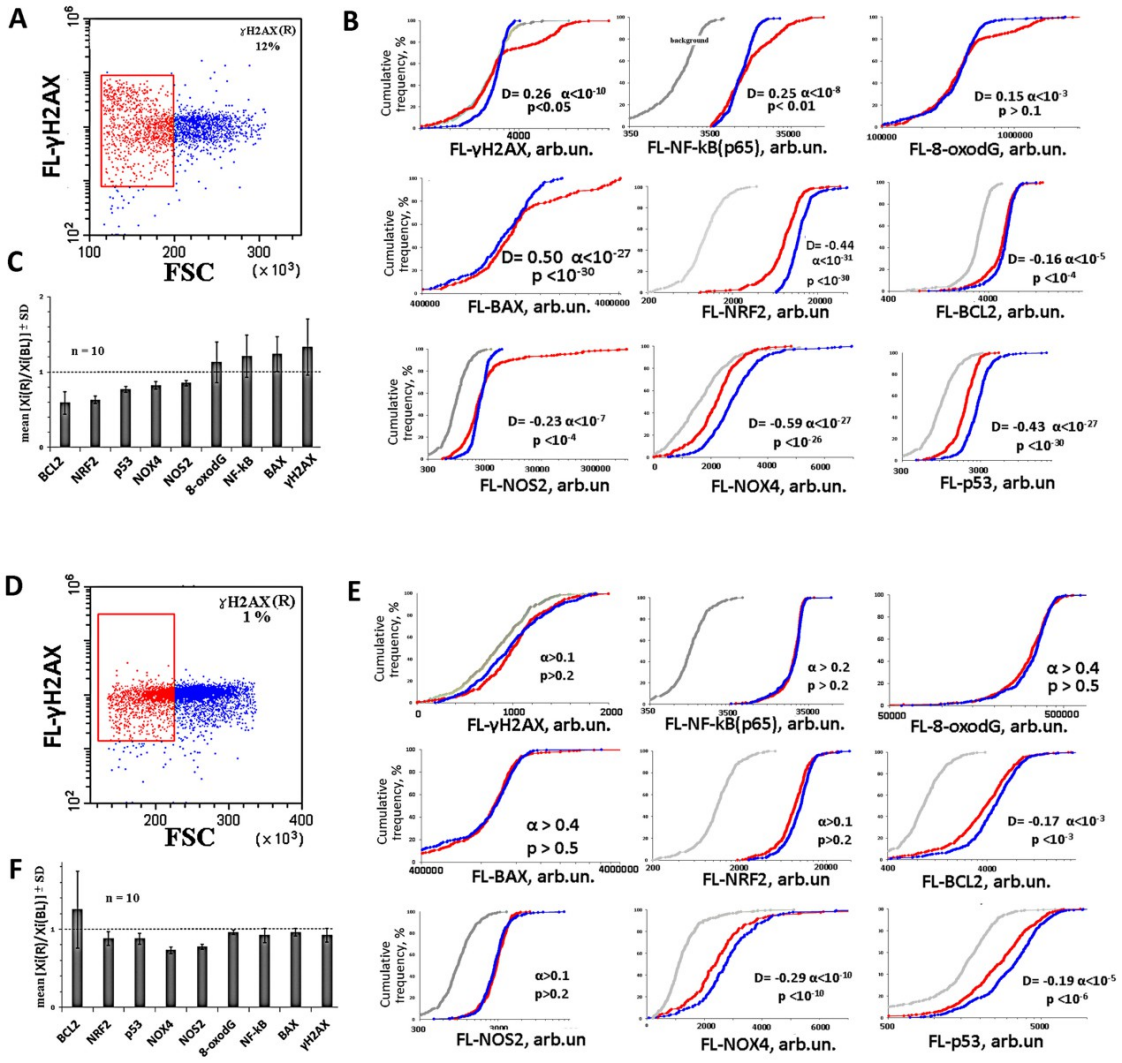
Analysis of the subpopulations of the HC lymphocytes with a high and low γH2AX(R) index value. (**A** and **D**) An example of the analysis of the population of lymphocytes. We divided the population into two subpopulations—the cells with relatively small FSC parameter values (red-fraction) and the rest (blue-fraction); (**B** and **E**) for each fraction, the cumulative distribution of several markers were determined separately. The analyzed marker is indicated along the X-axis. The significance of the observed differences was analyzed using the non-parametric Mann–Whitney test (p) and Kolmogorov–Smirnov test (D and α). (**C** and **F**) the ratio of the analyzed indices for red- and blue-subpopulations (mean ± SD for 10 lymphocyte samples).

Fig 7A and 7D show the analysis examples for two samples of lymphocytes with high and low γH2AX(R) index values. We found that the γH2AX(R) fraction cells have smaller sizes (FSC parameter) and are located mainly in the area indicated on the FSC-SSC plot by red color [Fig 7A]. Thus, we divided the cells into two subpopulations—the cells with relatively small FSC parameter values (red color, red-fraction) and the rest (blue color, blue-fraction). For each fraction, the levels of several markers were determined separately. The results for two typical samples are presented in the form of cumulative distributions of the experimental parameter values. The five markers described above (γH2AX, 8-oxodG, NOX4, BAX, BCL2 and NRF2) were analyzed. Additionally, the levels of proteins NF-kB(p65) (transcription factor regulating cytokine synthesis), p53 (transcription factor regulating the DNA damage response) and NOS2 (one of the oxidative stress inducers) were determined.

In HC samples with high γH2AX(R), the red fraction contained two types of cells. 20–30% of the red fraction cells contained abnormally high γH2AX, BAX, NF-kB(p65), NOS2, and 8-oxodG markers levels, and abnormally low NRF2 levels. In the HC samples with low γH2AX(R), the γH2AX, NOX4, NF-kB(p65), NOS2, 8-oxodG and NRF2 markers levels in the two fractions did not differ (Fig 7).

Thus, we found red-fraction heterogeneity in the HC samples population with high γH2AX(R) in terms of the markers content reflecting cellular oxidative stress response. High double-strand breaks number, high BAX and a low BCL2 and NRF2 levels [Fig 7C] may indicate the DNA damage repair slowdown. However, such cells remain in the population and are not lost during lymphocytes isolation from the blood. SZ cells samples, as a rule, did not contain the red-fraction of cells, which would differ significantly by the analyzed parameters values, reflecting the repair and apoptosis levels.

Fig 5C shows cfDNA concentration dependence on γH2AX(R). The maximum cfDNA concentrations were determined in the plasma of HC blood samples with low γH2AX(R) values for the isolated lymphocytes population. It can be assumed that in the blood of schizophrenia patients an increased DNA damage level in cells is combined with a more effective clearance of dead cells from the bloodstream (Fig 5D). The main sources of circulating cell-free DNA are the cells that have died.

## Discussion

In the present study, we analyzed the 8-oxodG, NOX4, ɣH2AX, NRF2, BAX and BCL2 levels in 60 control PBL samples and 100 PBL samples of the untreated SZ patients. 8-oxodG, NOX4, and BCL2 levels in the PBL in the SZ group were higher than those in the HC group. ɣH2AX protein level was increased in the subgroup with high 8-oxodG levels and decreased in the subgroup with low 8-oxodG levels. A positive correlation was found between 8-oxodG, ɣH2AX and BAX1 levels in the SZ group. NOX4 level in lymphocytes did not depend on the DNA damage markers values and BAX1 and BCL2 proteins levels.

The FCA revealed significant heterogeneity in terms of the DNA oxidation marker content in the SZ group (Fig 2).

### Subgroups SZ(8-oxodG<0.25)

64% of SZ PBL did not differ from the HC PBL by 8-oxodG level (Fig 2E). However, we found a significant difference between the SZ(8-oxodG<0.25) subgroups and the HC group in several other markers that characterize the response of cells to oxidative stress.

PBL in the SZ (8-oxodG <0.25) subgroups contain significantly less double-strand DNA break (marker γH2AX) than HC PBL (Fig 3F*a*). A decrease in the level of γH2AX in SZ(8-oxodG <0.25) PBL as compared to HC lymphocytes indicates the DNA repair systems activation in response to DNA damage.

The BAX/BCL2 ratio, characterizing the apoptosis level [Korsmeyer et al., 1993], was lower in the SZ(8-oxodG <0.25) subgroups than in the HC group (Fig 4G*b*).

Despite the low level of DNA oxidation in isolated lymphocytes, oxidative stress actually exists in the body of these patients. It is indicated by the increased concentrations of cfDNA in the plasma, which was studied in the same blood samples as PBL (Fig 5B). Plasma cfDNA concentrations in the SZ(8-oxodG<0.25) subgroup correlate with the DNA oxidation marker 8-oxodG level (Rs = 0.36, p = 0.003, N = 64) and the level of apoptosis marker BAX/BCL2 ratio (Rs = 0.32, p = 0.010, N = 64) in isolated lymphocytes.

Thus, in lymphocytes of the SZ (8-oxodG<0.25) subgroups, we observed the signs of a successful development of an adaptive response to oxidative stress, which includes the activation of DNA repair and antiapoptotic responses.

### Subgroups SZ(8-oxodG>0.25)

36% of SZ PBL (SZ-2 and SZ-4 subgroups) contained more DNA oxidation marker than the sample with the maximum 8-oxodG level in the HC group (Fig 2). Plasma concentrations of cfDNA in the SZ(8-oxodG>0.25) blood samples are significantly higher than in the plasma of SZ(8-oxodG<0.25) samples (D = 0.34, α = 0.008; p< 0.015). High level of DNA oxidation marker in lymphocytes and high DNA concentrations in plasma indicate a higher levels of oxidative stress and cell death in the body of these patients.

In the SZ (8-oxodG>0.25) lymphocytes, the level of ɣ-H2AX is increased compared to the HC group and SZ (8-oxodG<0.25) subgroup (Fig 3). In lymphocytes of this group, the amount of pro-apoptotic protein BAX is increased. However, the amount of the antiapoptotic protein BCL2 also increased (Fig 4). The BAX/BCL2 ratio in the SZ(8-oxodG>0.25) subgroup was significantly higher than in the SZ(8-oxodG<0.25) samples, but did not differ from the control values.

In the SZ-4 (8-oxodG>0.25; NOX4>0.35) subgroup, we found an increase in the amount of the factor NRF2 that regulates the antioxidant response. However, the ratio NRF2/NOX4 in this subgroup was significantly lower than in the control samples (Fig 6).

The aforementioned features of the SZ (8-oxodG> 0.25) PBL and plasma indicated that the ROS level in the patient’s body significantly exceeds the ability of the PBL to develop an effective adaptive response.

Patients in the SZ (8-oxodG>0.25) subgroups had a more severe disease, worser responded to the therapy and had a higher PANSS score than patients in the SZ (8-oxodG <0.25) subgroup (Table 1).

### High clearance of damaged cells in SZ patients

15% of the HC samples contained from 5 to 12% cells with relatively small sizes and high **ɣ-H2AX** levels [fraction ɣ-H2AX(R)]. The cells of this fraction also had high 8-oxodG and BAX levels and low NRF2, NOX4 and BCL2 levels (Fig 7). In remaining cells of these PBL samples, the content of ɣ-H2AX, 8-oxodG, and BAX markers was reduced. It can be assumed that in the HC subpopulations with high markers levels, the DNA repair processes and the antioxidant response are reduced. However, such cells are not eliminated from the population. Perhaps their stability is due to high NF-kB levels and low NOX4 levels. Cells with these features are much less common in SZ(8-oxodG<0.25) populations of lymphocytes (Fig 3). Possibly the response of SZ(8-oxodG<0.25) PBL to oxidative stress in the body involves more active elimination of cells subpopulation with unrepairable DNA damage.

Increased damaged cells clearance in the population of schizophrenia patients’ blood cells is indirectly reflected by the increased cfDNA concentrations in the plasma of blood samples from which the lymphocytes were isolated (Fig 5D). Despite the lower BAX/BCL2 index in SZ lymphocytes, much more fragments of cellular DNA circulate in the extracellular environment of blood, which indicates an increased level of cell death and increased clearance of dead cells from the population. DNA fragments of dead cells increase the cfDNA pool.

### NOX4 is a likely component of the adaptive response

One of the main findings of our study is increased *NOX4* gene expression in PBL of the SZ patients. According to recent studies, NOX4 plays a significant role in the development of several diseases [22]. NOX4 is the cell oxidoreductase, catalyzing the hydrogen peroxide formation. High levels of NOX4 in the cells can potentially be associated with high cellular DNA oxidation levels and DNA damage. However, we did not find any **NOX4** correlation with the **8-oxodG** index reflecting DNA oxidative modification (Rs = 0.08, p = 0.42, N = 100; Table 2) and with the **γH2AX index** reflecting DNA double-strand breaks (Rs = 0.18, p = 0.09, N = 100). SZ (NOX4>0.35) and SZ (NOX4<0.35) subgroups did not differ by the amount of 8-oxodG marker (p >0.3) and γH2AX marker (p>0.1, Fig 3). The SZ (8-oxodG<0.25) and (8-oxodG>0.25) subgroups also did not differ by the protein NOX4 amounts (p>0.1) (Fig 2E). The cell populations with low level of DNA oxidation and a low level of DNA fragmentation may contain significant NOX4 amounts. The SZ group included twenty one such PBL samples (SZ-3 subgroup). Conversely, the cell populations with a high level of DNA oxidation and a high level of DNA fragmentation may contain low NOX4 amounts (SZ-2 subgroup). The SZ group included twenty such PBL samples. Moreover, NOX4 level negatively correlates with pro-apoptotic **BAX** and **BAX/BCL2** ratio in HC group and positively correlates with anti-apoptotic BCL2 and NRF2 levels in SZ group (Table 2).

It can be speculatively assumed that *NOX4* gene over-expression in the lymphocytes of some SZ patients results from the development of an adaptive response improving cell survival under conditions of oxidative stress, which is aggravated during disease exacerbation. We discussed earlier that the response of schizophrenic lymphocytes to endogenous oxidative stress is very similar to the response of healthy donor cells to the low doses of ionizing radiation (LDIR) [20, 49]. Indeed, *NOX4* gene expression seemed to elevate significantly in both in differentiated and stem human cells within the first hours after LDIR exposure [50].

*NOX4* expression is thought to be necessary for the adaptive response induction. First, H_2_O_2_ and H_2_O_2_-generated ROS induce changes in nuclear chromatin conformation, resulting in alteration in the profiles of gene expression. Antioxidant-induced ROS inactivation disrupts changes in the chromatin transformation [51]. Earlier we showed that conformation of chromatin in the interphase nuclei of SZ patients lymphocytes is similar to that of chromatin in the nuclei of irradiated lymphocytes obtained from healthy donors. These changes are believed to indicate an adaptive response development [50].

Some investigators also tend to believe that NOX4 is an essential component of the adaptive response that promotes cell survival in an unfavourable environment. In some studies, inhibition of NOX4 activity may result in cell apoptosis [26–30]. NOX4 deficiency exacerbates the impairment of hippocampal neurogenesis due to chronic high fat diet. These data suggest that NOX4 could be a critical protein for protecting against neuronal disorders during chronic metabolic diseases [31]. It was also shown that Nox4 mediates protection against chronic load-induced stress in mouse hearts by enhancing angiogenesis [32].

### ROS production by NOX4 may accelerate the clearance of the damaged cells

Exuberant ROS production can induce cellular death in the subpopulations of the cells with extremely high NOX4 levels and reduced NRF2 levels (Fig 6E). In this way, the cells with very high levels of irreparable damage are eliminated from the population. The DNA of these dead cells increases the circulating cfDNA pool. Low NOX4 levels in some lymphocyte subgroups of healthy controls appear to be associated with poor elimination of defective cells due to low ROS production (Fig 7). The presence of a few cells with abnormally high marker levels in the PBL pool of healthy volunteers can reduce differences in some overall parameters (e.g., RNA level detected by PCR, Western blot analyses data) between the control and the patient groups. Possibly, additional stress, e.g., exercising, could accelerate the elimination of damaged cells from the circulation of healthy people.

In the subgroups of patients with high NOX4 levels in the lymphocytes (SZ-3 and SZ-4) cfDNA concentrations were significantly higher than in the subgroups with low NOX4 levels or in the control (Fig 5B). Therefore, high NOX4 expression in the PBL population seems to be associated with a more effective clearance of dead cells from the cell populations in the body and the appearance of a large number of DNA fragments of these cells in the blood (Fig 5D).

The use of antioxidants in the therapy of patients with schizophrenia is widely discussed in literature [15]. Data received by different authors vary considerably. In our view, inclusion of antioxidants in schizophrenia therapy should be considered individually for each patient. Excessive decline in NOX4-induced ROS production can block adaptive responses and increase cellular DNA damage. In addition, over-blocking of extracellular ROS by effective antioxidants can lead to the secondary oxidative stress associated with accumulation of NOX4 protein in the cytoplasm and nucleus within the late periods of the cellular response [52]. As a result, the number of damaged cells in tissues and organs, including brain, may significantly increase. It is possible that very high level of the DNA oxidation marker 8-oxodG in the patient’s lymphocytes is an indication for the antioxidants administration.

*The limitations of this study*. In our study, we specifically selected patients who had never (or for a long time) taken antipsychotic drugs. Potentially the therapy can significantly alter the DNA damage level and *NOX4* gene expression. In the future, it would be useful to compare NOX4 and 8-oxodG levels in the PBL obtained from newly diagnosed SZ patients before and after combined therapy with the ordinary used antipsychotics and antioxidants as well.

## Conclusion

A positive correlation was found between 8-oxodG, ɣH2AX and BAX1 levels in the SZ group. Significant *NOX4* gene expression was found in SZ patients’ lymphocytes, but NOX4 level in lymphocytes did not depend on the DNA damage markers values and BAX1 and BCL2 proteins levels. NOX4 protein is probably not a cause of the DNA damage.

## Abbreviations

FCA: flow cytometry analysis
HC: healthy control
mtDNA: mitochondrial DNA
PBL: a peripheral blood lymphocytes
PBMC: peripheral blood mononuclear cells
ROC analysis: “Receiver Operating Characteristic” curve analysis
SZ: schizophrenia
